# Electrosprayed Molybdenum Trioxide Aqueous Solution and Its Application in Organic Photovoltaic Cells

**DOI:** 10.1371/journal.pone.0106012

**Published:** 2014-08-22

**Authors:** Katsumi Suzuki, Takeshi Fukuda, Yingjie Liao

**Affiliations:** Department of Functional Materials Science, Graduate School of Science and Engineering, Saitama University, Saitama, Japan; RMIT University, Australia

## Abstract

A molybdenum trioxide thin film with smooth surface and uniform thickness was successfully achieved by an electrospray deposition method using an aqueous solution with a drastically low concentration of 0.05 wt%. Previous papers demonstrated that an additive solvent technique is useful for depositing the thin film by the electrospray deposition, and the high vapor pressure and a low surface tension of an additive solvent were found to be important factors. As a result, the smooth molybdenum trioxide thin film was obtained when the acetonitrile was used as the additive solvent. Furthermore, the vapor pressure of acetone is much higher than that of aqueous solution, and this indicates that the acetone is easily evaporated after spraying from the glass capillary. By optimizing a concentration of acetone in the molybdenum aqueous solution, a minimum root mean square roughness of the MoO_3_ thin film became 3.7 nm. In addition, an organic photovoltaic cell was also demonstrated using the molybdenum trioxide as a hole transport layer. Highest photoconversion efficiency was 1.72%, a value comparable to that using conventional thermal evaporation process even though the aqueous solution was used for the solution process. The photovonversion efficiency was not an optimized value, and the higher value can be achieved by optimizing the coating condition of the active layer.

## Introduction

Organic thin films can be formed by several solution processes such as conventional spin-coating, inkjet printing, gravure printing, screen printing, doctor blading, and ultrasonic spraying methods [1], [2]. Nowadays, several kinds of organic thin film devices, including organic light-emitting diodes (OLEDs) [3], organic photovoltaic cells (OPVs) [4]–[6], organic image sensors [7], and organic thin-film transistors [8], [9] have been investigated for future printed/ambient electronics applications. This is because specific organic molecules exhibit high photoluminescence quantum efficiency [10], high optical-to-electrical conversion efficiency, selective absorption band in the visible wavelength region [11], and high carrier mobility compared to conventional inorganic materials [12].

Among the reported solution processes, the electrospray deposition (ESD) method has gained interest as a novel coating process for organic thin-film devices. Several theoretical approaches were investigated to understand the spray mechanism [13]–[15], and organic thin films are easily deposited by the ESD. However, a disadvantage of the ESD process is a difficulty to form smooth organic thin film compared to other solution process. Even though the high dielectric constant of solutions is an important factor for the ESD process [16], most of the organic semiconductor materials cannot be dissolved in polar solvents. Therefore, an efficient electrospray was not realized owing to the low dielectric constant of normal solutions containing organic semiconductors. Recently, Ju *et al*. demonstrated that a smooth surface of the organic thin film could be achieved by the adding solvent with a high dielectric constant [17]. Thus far, OLEDs [18], photoconductive devices [19], thin-film transistors [20], and OPVs [21]–[23] have been fabricated using the ESD process. In addition, other special advantages of the ESD process include the possibility for multilayer structures [24], [25] and controlled molecular alignment/crystallinity/segregation of the thin films and [26]–[29]. Therefore, higher device performances are expected even though it involves a simple experimental setup without a vacuum condition, which could enable the realization of drastically low fabrication cost compared to the conventional thermal evaporation process.

Another most important advantage of the ESD process is that the dilute solution with a concentration of less than 1 mg/mL can be used to form organic thin films. In general, the concentration of the organic solution needs approximately 10 mg/mL for the OPV device owing to the thick organic active layer. On the other hand, several solution processes solve the above-mentioned problem [30]–[32]; however, the vacuum chamber is needed for the deposition process. In the ESD process, the viscosity range is drastically larger compared to other solution processes such as the spin-coating process, and the thickness of the deposited thin film can be controlled by changing the deposition time. Recently, MoO_3_ aqueous solution has been used for the fabrication of OLEDs and OPVs [33]–[35]; however, the maximum concentration of the MoO_3_ aqueous solution is rather low, resulting in a difficulty in forming the thin film by conventional solution processes. Therefore, the MoO_3_ was difficult to be formed by the conventional solution process, and we investigated the optimized fabrication process for the MoO_3_ thin film by the ESD process using an aqueous solution. In addition, the MoO_3_ thin films have been widely used as hole transport layers for OLEDs and OPVs in previous studies [36], [37]. Because the energy level of MoO_3_ is suitable for the efficient hole transport between an adjacent organic layer and an indium tin oxide anode. In addition, the electronic properties of MoO_3_ is easily manipulated through ionic doping and other approaches, and this leads the reasonable charge carrier mobility due to the quantum confinement effect [38], [39].

In this paper, the MoO_3_ aqueous solution was electrosprayed using the additive solvent technique, and several kinds of additive solvents such as acetone, acetonitrile, *N*,*N*-dimethylformamide (DMF), and dimethyl sulfoxide (DMSO) were used to improve the surface morphology/uniformity of the MoO_3_ thin film. Furthermore, OPVs with the MoO_3_ hole transport layer were estimated for comparing the conventional spin-coating process. A main purpose of this study is to find the optimized deposition condition of the MoO_3_ thin film by the ESD. Therefore, the fabrication process of active layer was not optimized, and we discussed the relationship between the fabrication condition of MoO_3_ layer and the OPV performance.

## Materials and Methods

**Figure 1:**
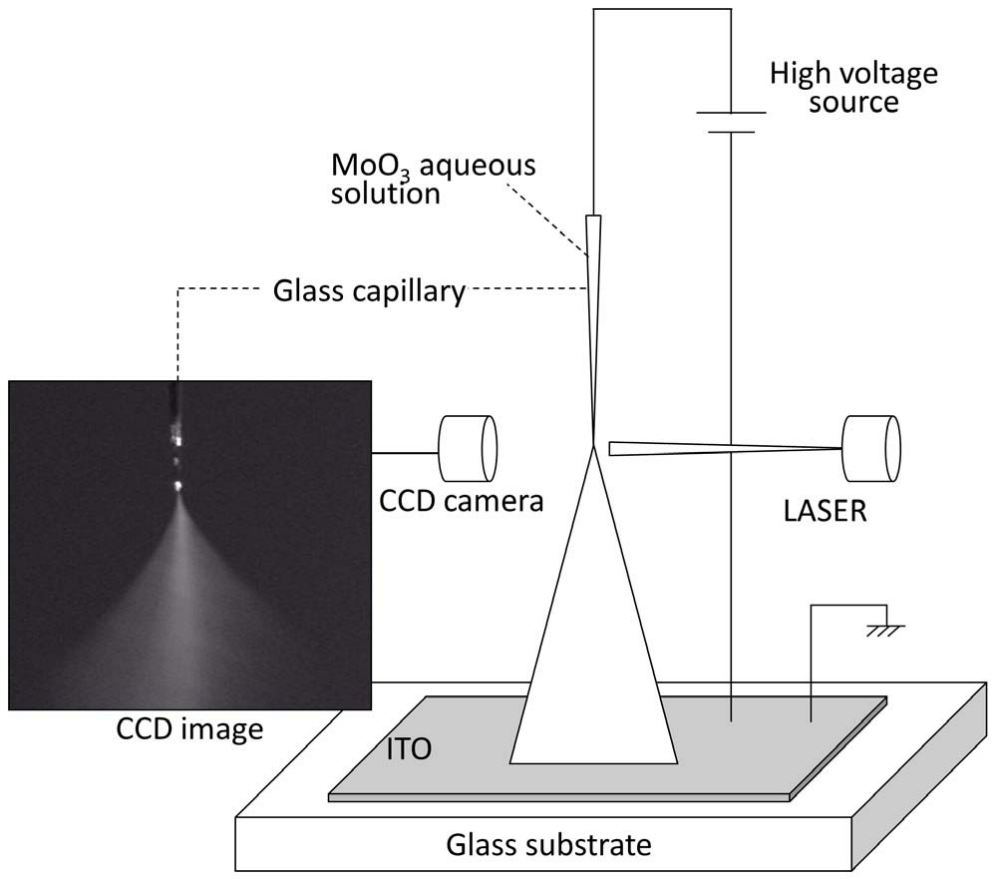
Schematic configuration of the ESD setup.

A schematic configuration of the ESD setup is shown in Fig. 1 [22], [23]. A glass capillary was fabricated using a puller (PC-10, Narishige) and a microforge (MF-900, Narishige). The inner diameter of the glass capillary was approximately 50 µm. A positive high voltage was applied to a copper wire in the MoO_3_ aqueous solution using a high-voltage source (ETM3-20K01PN1, Element). An earthed line was also connected to a patterned indium tin oxide (ITO) layer on top of the glass substrate. In addition, the spray condition was imaged using a charge coupled device (CCD) camera placed near the tip of the glass capillary, and the visible laser was irradiated the tip of the glass capillary.

A preparation of used solutions is as follows. The MoO_3_ powder was dissolved in a pure water at a concentration of 0.05 wt%, and stirred for 24 h at a room temperature. In addition, an additive solvent was also added into the resulting aqueous solution. In this study, we used acetone, acetonitrile, DMF, and DMSO as the additive solvents, and they were already used as the additives for the solution containing organic semiconductor materials to fabricate the OPVs [22], [23]. Next, the resulting solution was passed through a filter with a hole diameter of 450 nm. For the organic active layer, 12 mg of poly(3-hexylthiophene) (P3HT) and 10 mg of (6,6)-phenyl-C61-butyric acid methyl ester (PCBM) were mixed into 1 mL of *o*-dichlorobenzene (*o*-DCB). Finally, the P3HT:PCBM solution was also passed through a filter.

An OPV was fabricated in a subsequent process. In this research, we want to investigate the effect of the MoO_3_ layer fabricated by the ESD; therefore, conventional OPV devices were fabricated. First, an ITO-coated glass substrate was cleaned in solvent and deionized water under ultrasonic waves, and was treated with ultraviolet ozone cleaning for 20 min. Then, the MoO_3_ aqueous solution was electrosprayed on the ITO-coated glass substrate. The distance between the glass capillary and the substrate was 5 cm and the deposition time was 10 min. The sample was then annealed at 250°C for 10 min. After spin-coating the P3HT:PCBM layer at a rotation speed of 1000 rpm for 1 min, an Al electrode was thermally evaporated in a vacuum deposition chamber. Finally, the sample was annealed at 140°C for 25 min under nitrogen atmosphere. The thicknesses of the P3HT:PCBM and Al layers was 70 and 130 nm, respectively.

The surface tension of the MoO_3_ solution was measured by a pendant drop method. The surface roughness/uniformity was estimated using an atomic force microscope (AFM; SPA-300, Seiki) and a scanning electron microscope (SEM; S-4800, Hitachi Science System). The current-density-voltage characteristics of the OPV were measured using a Keithley series 2400 digital source meter under the standard solar spectrum (AM 1.5G, 100 mW/cm^2^).

## Results and Discussion

**Figure 2:**
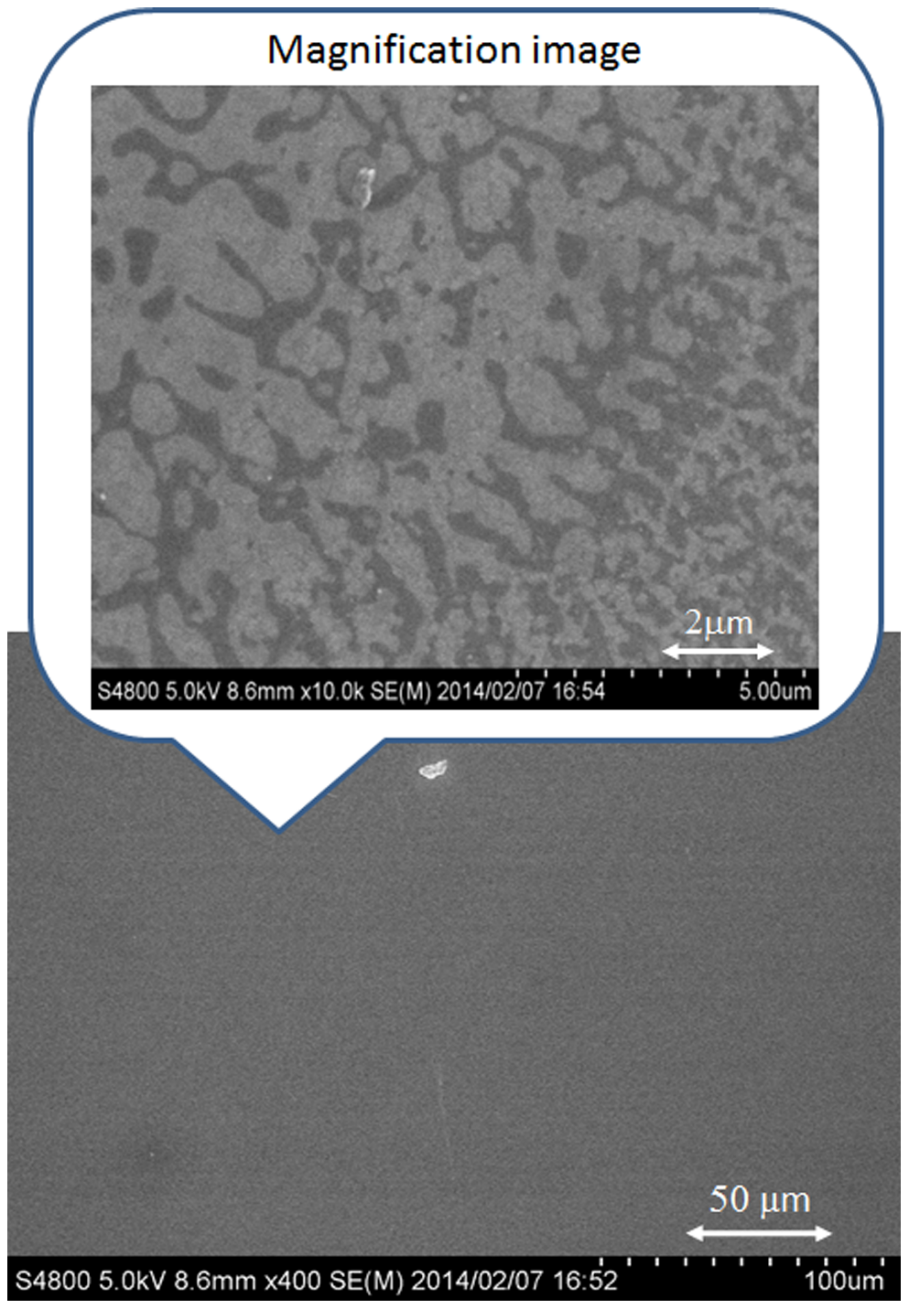
SEM image of the MoO_3_ thin film fabricated by the spin-coating process. The concentration of MoO_3_ aqueous solution was 0.05 wt%.

A typical SEM image of the MoO_3_ thin film, which was spin-coated on a glass substrate as a reference, is shown in Fig. 2. The smooth MoO_3_ layer seems to be coated by the spin-coating process in the SEM images; however, the uncoated regions were observed at the high-magnification SEM image shown in the inset of Fig. 2. The black regions in the SEM image indicate a lack of MoO_3_ layer, demonstrating that a uniform MoO_3_ thin film could not be realized. This is because the concentration of the MoO_3_ aqueous solution was 0.05 wt%, and it is drastically lower than that used for the conventional solution process. This result indicates that conventional spin-coating is not sufficient to form a uniform MoO_3_ thin film, and this MoO_3_ thin film is not suitable for the hole transport layer in the OPV.

**Figure 3:**
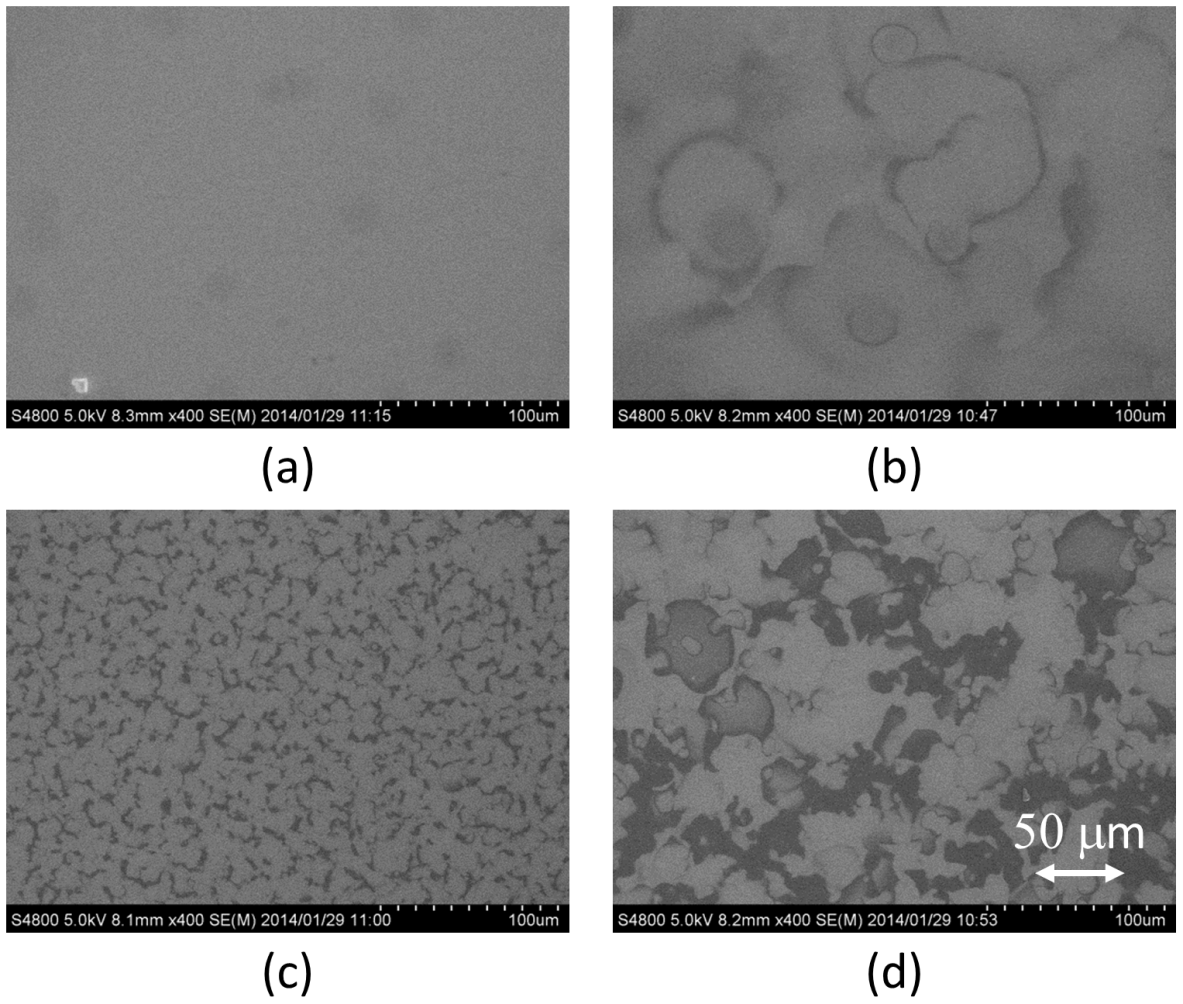
SEM images of MoO_3_ thin films fabricated by the ESD process. Additive solvents (20 vol%) were (a) acetone, (b) acetonitrile, (c) DMF, and (d) DMSO.

Figure 3 shows SEM images of MoO_3_ thin films fabricated by the ESD process with different additive solvents. The concentration of the additive solvent was fixed at 20 vol%, and (a) acetone, (b) acetonitrile, (c) DMF, and (d) DMSO were used. In the cases of DMF and DMSO, large water marks with a diameter of several 10 nm's were observed in the SEM images. Previous studies demonstrated that the dielectric constant of the additive solvent is an important factor for the ESD process to spread the spray diameter [16]; however, the pure water has much higher dielectric constant than the used organic solvents such as acetone, acetonitrile, DMF, and DMSO. This fact seems to cause the efficient electrospray for the aqueous solution; however, the surface tension of the solution is also an important parameter to discuss the electrospray phenomena. The Rayleigh limit (*Q_R_*) of the ESD is the charge amount of the droplet, and is given by the following equation [40], [41]:
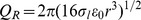
where σ_l_ is the surface tension, ε_0_ is the dielectric constant of the vacuum, and r is the diameter of the droplet. This indicates that that the low surface tension causes the low Rayleigh limit, resulting in the efficient fission process at the tip of the glass capillary.

In the SEM images as shown in Fig. 3, the smoothest MoO_3_ thin film was obtained when acetone was used as the additive solvent. The surface tension of acetone is 23.3 mN/m at a room temperature, lower than that of pure water (72.8 mN/m). Furthermore, the surface tension of the MoO_3_ aqueous solution was estimated as 44.0 mN/m after mixing acetone. This fact indicates that the Rayleigh limit became lower compared to the MoO_3_ aqueous solution by adding acetone, resulting in the efficient electrospray. Therefore, small droplets were continuously generated from the tip of the glass capillary [42], and the smooth MoO_3_ thin film was formed as shown in Fig. 3(a). On the other hand, the surface tensions of other additives are DMF (35.2 mN/m) and DMSO (43.5 mN/m), respectively, and they are much higher than that of acetone. The estimated surface tensions of mixed MoO_3_ aqueous solutions were 51 and 60 mN/m when DMF and DMSO were used as additives, respectively. These high surface tensions considered to cause the large droplet size, resulting in the many aggregated structure as shown in SEM images.

In general, the low vapor pressure of the droplet corresponding to the slow evaporation speed affects the surface morphology of the organic thin film due to the coffee stain phenomena [43]–[45]. Therefore, the vapor pressure of the additive solvent is also an important parameter for the surface roughness of MoO_3_ thin film. The vapor pressures of DMF (0.3 kPa at 20°C) and DMSO (0.059 kPa at 20°C) are rather lower than that of pure water (2.3 kPa), resulting in the low evaporation speed of the droplet. In this process, MoO_3_ aqueous droplets are continuously deposited, but the low vapor pressure of the additive solvent causes organic solvent to remain on the substrate after depositing the droplet. E. Ozden-Yenigun demonstrated that the drying process of the thin film was influenced by (i) the boundary layer resistance to solvent transport into vapor and (ii) the internal diffusion resistance to solvent loss [46]. In addition, the slow evaporation speed of the droplet occurs the main resistance to solvent loss, resulting in the free movement of solvent in the droplet. On the other hand, the surface tension of acetonitrile is 19.1 mN/m, resulting in the low surface tension of MoO_3_ aqueous solution (43.1 mN/m) even though the rough surface was observed in the SEM image shown in Fig. 3(b). A possible explanation is that the lower vapor pressure of acetonitrile than that of acetone caused the coffee stain effect, resulting in the observed many water marks. On the other hand, the vapor pressure of acetone is 24.7 kPa at 20°C, and most of the acetone was efficiently evaporated before reaching the substrate owing to the low boundary layer resistance between the droplet and the air. These results indicate that the high vapor pressure and the low surface tension are important factors for the MoO_3_ aqueous solution for the ESD.

**Figure 4:**
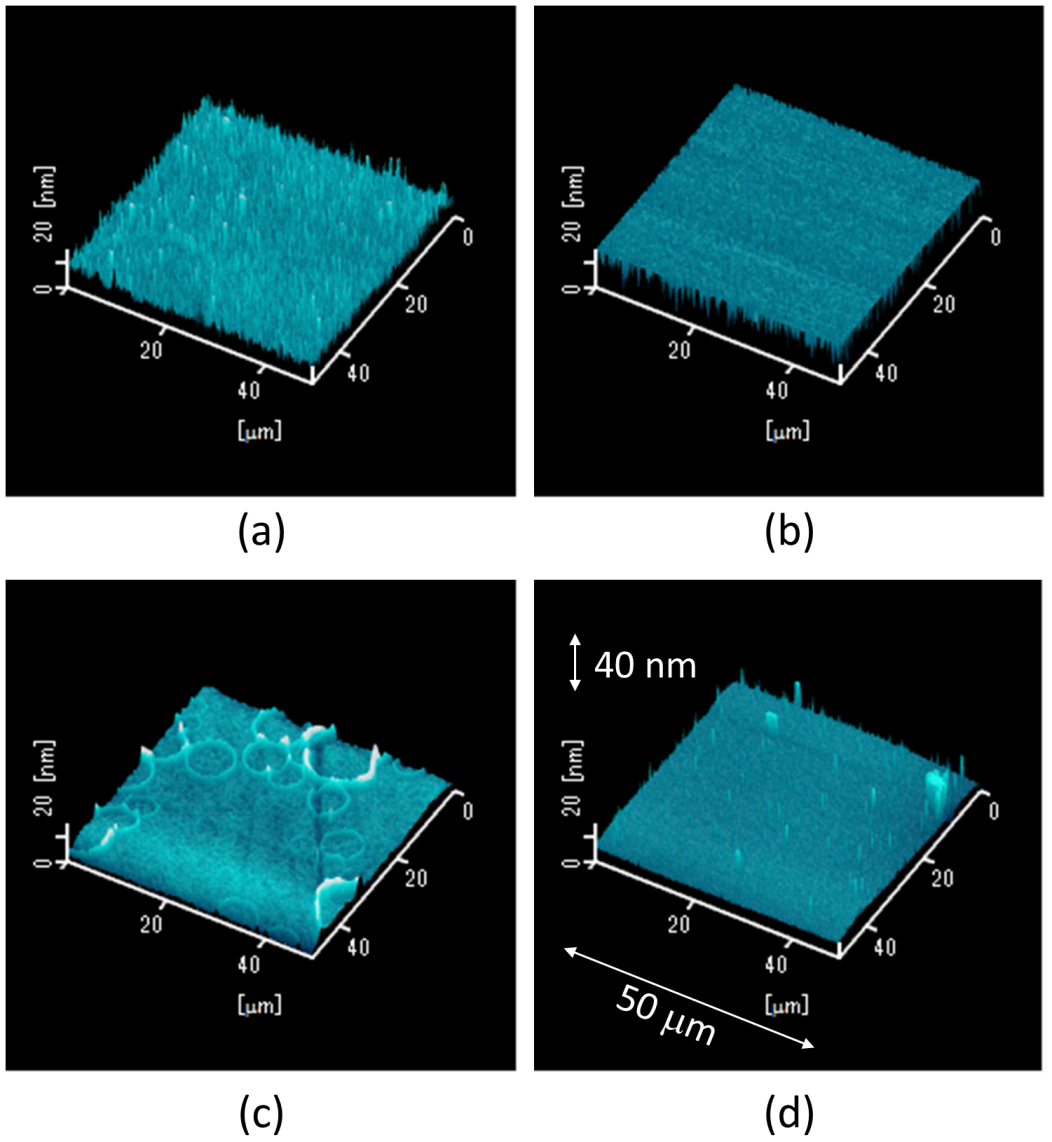
AFM images of MoO_3_ thin films fabricated by the ESD process. Additive solvents (20 vol%) were (a) acetone, (b) acetonitrile, (c) DMF, and (d) DMSO.

Figure 4 shows AFM images of MoO_3_ thin films deposited by the ESD process with different additive solvents, which were (a) acetone, (b) acetonitrile, (c) DMF, and (d) DMSO. The specific water mark was observed only in Fig. 4(c), and this sample was fabricated using DMF as the additive solvent. This result can be also explained by the low vapor pressure of DMF as in the SEM image. Since the scanning range is 50 µm ×50 µm for the AFM measurement, the water marks were not observed in Figs. 4(b) and 4(d) even though the SEM images showed nonuniform MoO_3_ thin films. The estimated root mean square (RMS) roughness was 4.3 nm in Fig. 4(a), and the smooth MoO_3_ thin film is smooth enough to coat an organic active layer (P3HT:PCBM). Anyway, the AFM image also indicates that acetone is the suitable solvent for depositing the MoO_3_ aqueous solution by the ESD process.

**Figure 5:**
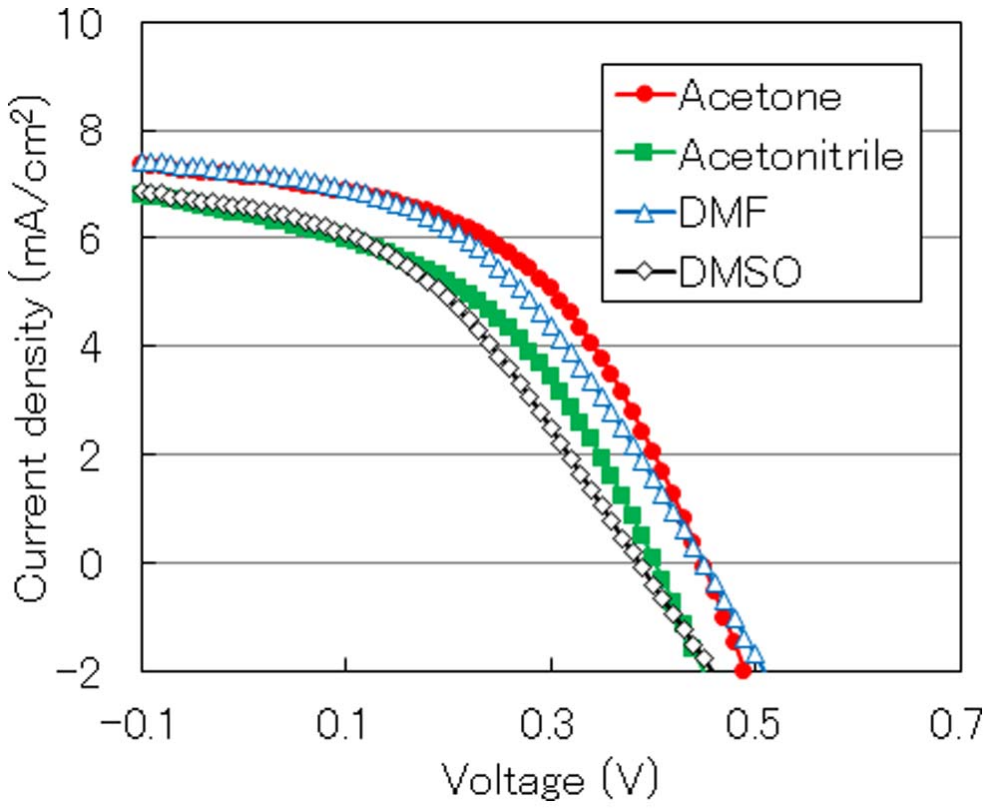
Current-density-voltage characteristics of OPVs with MoO_3_ layers fabricated using different additive solvents.

Figure 5 shows current-density-voltage characteristics of OPVs comprising the MoO_3_ thin film fabricated by the ESD process. Here, acetone, acetonitrile, DMF, and DMSO were used as additive solvents to deposit the MoO_3_ layer. In addition, Table 1 summarizes the photovoltaic characteristics: photoconversion efficiency (PCE), fill factor (FF), short-circuit current density (J_sc_), and open-circuit voltage (V_oc_) estimated from Fig. 5. In general, the FF value is influenced by the resistance component between the P3HT:PCBM and the MoO_3_ layers, and the increased FF is considered to be caused by the low carrier recombination probability owing to the smooth MoO_3_ layer when acetone was used as the additive solvent [47]. As a result, the highest PCE of 1.52% was achieved by using acetone as the additive solvent. Therefore, we investigated the effect of acetone concentration to improve the photovoltaic performance.

**Table 1:** PCE, FF, J_sc_, and V_oc_ of OPVs fabricated using different additive solvents.

Additive solvent	PCE (%)	FF	J_sc_ (mA/cm^2^)	V_oc_ (V)
acetone	1.52	0.48	7.14	0.45
acetonitrile	1.13	0.44	6.45	0.40
DMF	1.37	0.43	7.23	0.45
DMSO	0.989	0.39	6.58	0.39

**Figure 6:**
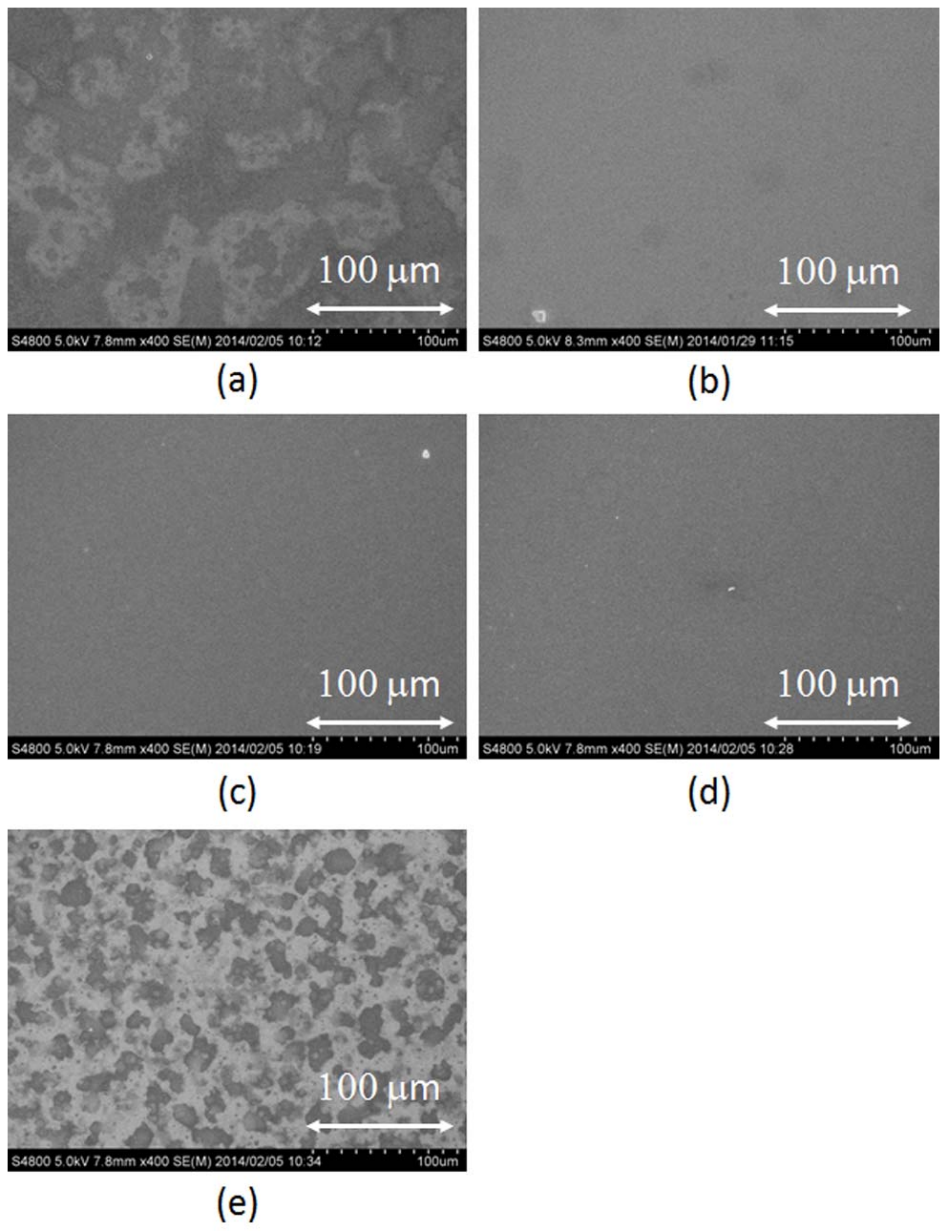
SEM images of MoO_3_ thin films fabricated with different concentrations of acetone. Concentrations ranged as (a) 0, (b) 20, (c) 30, (d) 40, and (e) 50 vol%.

The OPV performance and the surface morphology indicate that acetone is the suitable solvent for depositing the MoO_3_ aqueous solution by the ESD process. Therefore, we investigated the optimized concentration to improve the OPV performance. Figure 6 shows SEM images of MoO_3_ thin films fabricated using different concentrations of acetone: (a) 0, (b) 20, (c) 30, (d) 40, and (e) 50 vol%. A uniform film was not observed in the case of no additive solvent (pure water only), as shown in Fig. 6(a). In addition, the MoO_3_ thin film became drastically nonuniform at the concentration of 50 vol% (Fig. 6(e)). This is because the high concentration of acetone corresponds to the low concentration of MoO_3_ aqueous solution, which leads to the nonuniform MoO_3_ thin film. On the other hand, uniform MoO_3_ thin films were obtained at a concentration range from 20–40 vol%. Therefore, we can conclude that the optimum concentration of acetone was from 20–40 vol% to form a uniform MoO_3_ thin film. This result indicates that the acetone is useful additive solvent to form a smooth and uniform MoO_3_ thin film using an aqueous solution.

**Figure 7:**
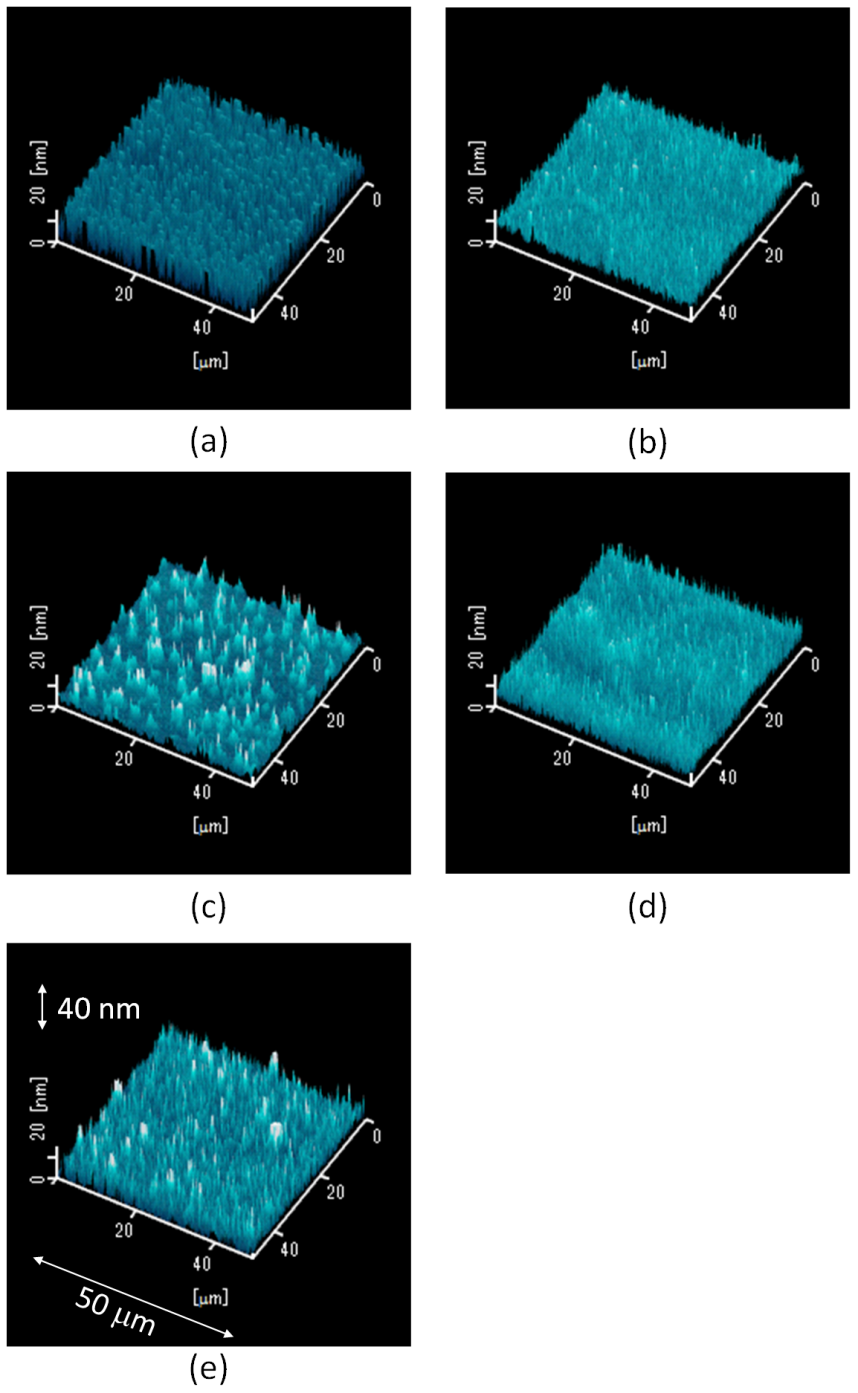
AFM images of MoO_3_ thin films fabricated using different concentrations of acetone. Concentrations were (a) 0, (b) 20, (c) 30, (d) 40, and (e) 50 vol%.

Figure 7 shows AFM images of MoO_3_ thin films deposited using different concentrations of acetone ranged from 0 to 60 wt%. The AFM images of all the samples were almost the same; this indicates that a smooth thin film can be obtained in the small area (50 µm ×50 µm). The RMS roughness of MoO_3_ thin films were 4.3, 4.1, 4.3, and 4.4 nm for the concentration of 30, 40, 50, and 60 vol%, respectively. The RMS roughness of several nm's was observed in Figs. 7(b)–7(d), corresponding to a uniform MoO_3_ thin film fabricated with concentrations ranging from 20–40 vol%.

**Figure 8:**
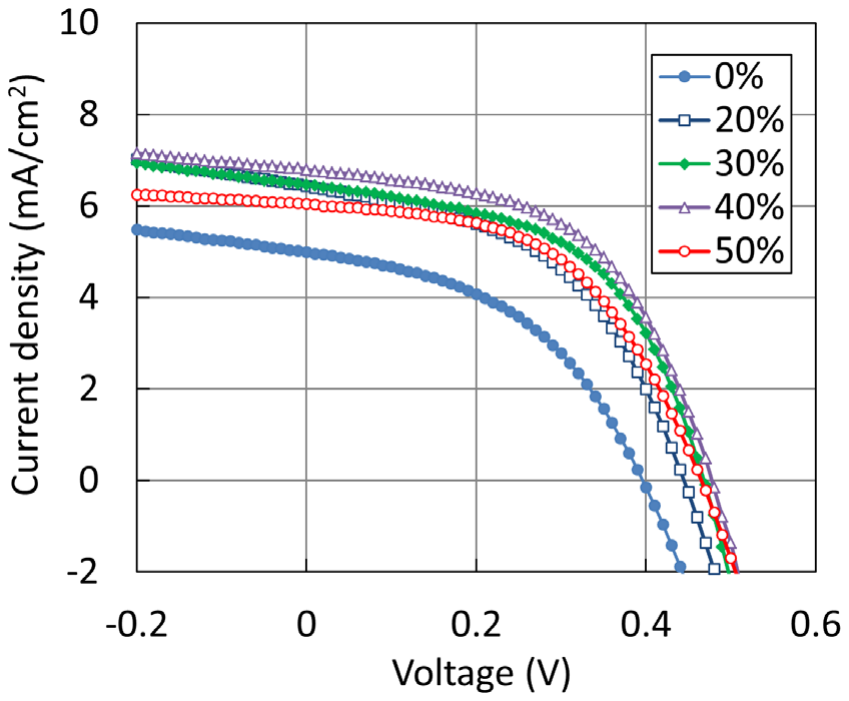
Current-density-voltage characteristics of OPVs incorporating MoO_3_ layer fabricated using different concentrations of acetone.

**Figure 9:**
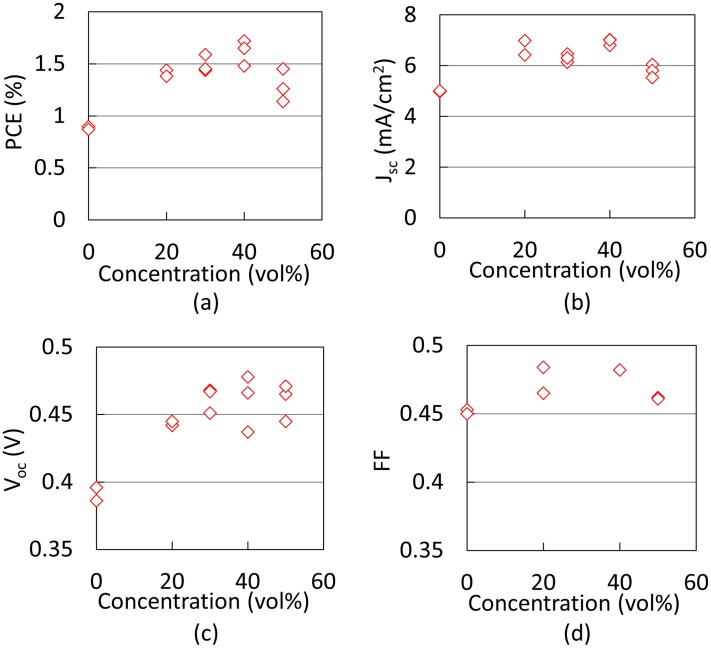
OPV characteristics as a function of acetone concentration.

Figure 8 shows current-density-voltage characteristics of OPVs incorporating the MoO_3_ layers, which were deposited by the ESD process using different concentrations of acetone. In addition, Fig. 9 shows (a) PCE, (b) FF, (c) J_sc_, and (d) V_oc_ values as a function of acetone concentration in the MoO_3_ aqueous solution. There devices were fabricated to estimate the fluctuation of device performance. The highest PCE of 1.72% was obtained at a concentration of 40 vol%, and this is considered to be realized due to the smallest RMS roughness of MoO_3_ thin film calculated from Fig. 9(d). In addition, the MoO_3_ layer was deposited by the conventional thermal evaporation process as a reference device and the PCE of the reference device was 2.04%. The PCE was relatively low compared to reported values; however, this is due to the nonoptimized fabrication condition of the active layer. This indicates that the fabrication condition of the MoO_3_ layer was well optimized by the above-mentioned experimental results.

## Conclusion

We successfully achieved uniform and flat MoO_3_ thin films deposited by the ESD process using aqueous solution with an additive. We found that the high vapor pressure and the low surface tension are important parameters to form the smooth MoO_3_ thin film. The uniformity of the MoO_3_ layer resulted in high OPV performance. This result will lead us to realize organic-inorganic thin film devices using the ESD process in the near future.
